# Gastrotomy in Internal Strangulation

**Published:** 1872-12

**Authors:** 


Gastrotomy in Cases of Internal Strangulation.—Dr. Delaporte, in an inaugural thesis just published in Paris, having carefully compiled and investigated a large number of cases bearing upon the above subject, comes to the following conclusions: Gastrotomy, far from being an invariably fatal operation, has afforded as numerous successful results as other major operations. Gastrotomy, which is pointed out by theory as the best surgical treatment of internal strangulation in the majority of its forms, should therefore hold its place in practice. Gastrotomy, which offers as many chances of success as enterotomy, and which gives more complete results of cure, should always be preferred to enterotomy in cases where some doubt exists as to the cause of strangulation or the choice of an operation. Gastrotomy, when once seen to be necessary and decided upon, must be performed as soon as the symptoms leave no doubt as to the existence of internal strangulation, and as early as possible in order to frustrate the development of peritonitis, the formation of adhesions, gangrene of the intestine, and exhaustion of the patient. The opening of the abdomen must be made in the mesial line, and largely, so as to permit free access to the cause of the strangulation. If the strangulation cannot be reduced the operation must be terminated by opening the bowel in the most favorable situation, and the establishment of an artificial anus through the primary incision. Gastrotomy would thus be performed.—*London Lancet.*

Tracheotomy with the "Galvano-Caustic Knife."—M. Verneuil, of Paris, in a case where he desired to open the trachea, employed the "galvano-caustic knife," the blade of which was heated to a dull red by means of a caustic battery, and the operation performed as with an ordinary knife. The pain, he contends, was not so great as with the ordinary knife, and there was but little hœmorrhage. The wound did well.

